# Preclinical Testing Oncolytic Vaccinia Virus Strain GLV-5b451 Expressing an Anti-VEGF Single-Chain Antibody for Canine Cancer Therapy

**DOI:** 10.3390/v7072811

**Published:** 2015-07-20

**Authors:** Marion Adelfinger, Simon Bessler, Alexa Frentzen, Alexander Cecil, Johanna Langbein-Laugwitz, Ivaylo Gentschev, Aladar A. Szalay

**Affiliations:** 1 Department of Biochemistry, Theodor-Boveri-Institute, University of Wuerzburg, D-97074 Wuerzburg, Germany; E-Mails: marion.adelfinger@biozentrum.uni-wuerzburg.de (M.A.); simon.bessler@stud-mail.uni-wuerzburg.de (S.B.); alexander.cecil@biozentrum.uni-wuerzburg.de (A.C.); johanna.langbein@biozentrum.uni-wuerzburg.de (J.L.-L.); 2 Genelux Corporation, San Diego Science Center, San Diego, CA 92109, USA; E-Mail: afrentzen@genelux.com; 3 Department of Bioinformatics, Theodor-Boveri-Institute, University of Wuerzburg, Biocenter, D-97074 Wuerzburg, Germany; 4 Department of Radiation Oncology, Rebecca & John Moores Comprehensive Cancer Center, University of California, San Diego, La Jolla, CA 92093, USA

**Keywords:** oncolytic virus, cancer, canine cancer cell lines, antibody production, angiogenesis, canine soft tissue sarcoma (CSTS), canine cancer therapy

## Abstract

Virotherapy on the basis of oncolytic vaccinia virus (VACV) strains is a novel approach for canine cancer therapy. Here we describe, for the first time, the characterization and the use of VACV strain GLV-5b451 expressing the anti-vascular endothelial growth factor (VEGF) single-chain antibody (scAb) GLAF-2 as therapeutic agent against different canine cancers. Cell culture data demonstrated that GLV-5b451 efficiently infected and destroyed all four tested canine cancer cell lines including: mammary carcinoma (MTH52c), mammary adenoma (ZMTH3), prostate carcinoma (CT1258), and soft tissue sarcoma (STSA-1). The GLV-5b451 virus-mediated production of GLAF-2 antibody was observed in all four cancer cell lines. In addition, this antibody specifically recognized canine VEGF. Finally, in canine soft tissue sarcoma (CSTS) xenografted mice, a single systemic administration of GLV-5b451 was found to be safe and led to anti-tumor effects resulting in the significant reduction and substantial long-term inhibition of tumor growth. A CD31-based immuno-staining showed significantly decreased neo-angiogenesis in GLV-5b451-treated tumors compared to the controls. In summary, these findings indicate that GLV-5b451 has potential for use as a therapeutic agent in the treatment of CSTS.

## 1. Introduction

Cancer is the major cause of canine death in both developed and developing countries [1]. Incidence of cancer ranges from 1% to 2% in the canine population and currently accounts for about half of the deaths in dogs older than 10 years [2,3,4]. The major treatment options for canine cancers include surgery, radiation therapy, chemotherapy, hyperthermia and photodynamic therapy. Despite progress in the diagnosis and treatment of advanced canine cancer, overall treatment outcome has not substantially improved in the past. Therefore, the development of new therapies for advanced canine cancer has a high priority. One of the most promising novel cancer therapies is oncolytic virotherapy. This method is based on the capacity of oncolytic viruses (OVs) to preferentially infect and lyse cancer cells without causing excessive damage to surrounding normal tissue. Several oncolytic viruses including various human and canine adenoviruses, canine distemper virus and vaccinia virus strains have been successfully tested for canine cancer therapy in preclinical settings (for reviews see [5,6]. However, in contrast to human studies, the clinical trials with oncolytic viruses for canine cancer patients are just at the beginning.

In the present study, we evaluated the oncolytic potential of the new recombinant oncolytic vaccinia virus GLV-5b451 expressing the anti-VEGF single-chain antibody (scAb) GLAF-2 against a panel of four different canine cancer cell lines. GLV-5b451 was derived from the oncolytic vaccinia virus LIVP 6.1.1 [7] by inserting the glaf-2 gene [8] encoding the GLAF-2 antibody under the control of the vaccinia virus synthetic early-late (SEL) promoter [9] into the *J2R* (encoding thymidine kinase) locus. VEGF or VEGF-A plays a critical role in promoting tumor angiogenesis. It was shown that overexpression of VEGF correlated well with tumor malignancy, as well as with a poor prognosis for the canine cancer patients [10,11,12,13]. Therefore, several anti-VEGF strategies have been developed for the treatment of different cancer patients [14,15,16]. We have already demonstrated that VACV expressing anti-VEGF antibodies exhibited significant reduction of tumor growth in canine xenografts and enhanced inhibition of angiogenesis in comparison to control animals [17].

Here, we analyzed the oncolytic effects of GLV-5b451 and the virus-associated anti-VEGF GLAF-2-antibody production in a panel of four different canine cancer cell lines (MTH52c, ZMTH3, CT1258 and STSA-1) and in a canine soft tissue sarcoma (CSTS) xenograft model.

## 2. Materials and Methods

### 2.1. Ethics Statement

All mice animal experiments were carried out in accordance with protocols approved by the Institutional Animal Care and Use Committee (IACUC) of Explora Biolabs (San Diego, CA, USA; protocol number: EB11-025) and/or the government of Unterfranken, Germany, according to the German Animal Welfare Act (TierSchG) (permit numbers: 55.2-2531.01-17/08 and 55.2-2531.01-24/12).

The MTH52c, ZMTH3, and CT1258 cell lines were obtained from Dr. I. Nolte (University of Veterinary Medicine, Hannover, Germany). The MTH52c is derived from a malignant small-cell canine carcinoma [18,19]. ZMTH3 is an immortalized canine mammary pleomorphic adenoma cell line [20,21]. The canine prostate carcinoma cell line CT1258 has been described previously [22,23]. African green monkey kidney fibroblasts (CV-1) cell line was provided by American Type Culture Collection (ATCC).

The STSA-1 cell line was obtained from Dr. A. MacNeill (University of Colorado, USA) and was derived from a canine patient with a low grade II soft tissue sarcoma [17,24].

### 2.2. Cell Culture

Cells were cultured in Dulbecco’s Modified Eagle’s Medium (DMEM) supplemented with antibiotic-solution (100 U/mL penicillin G, 100 units/mL streptomycin) and 10% fetal bovine serum (FBS; GE Healthcare/PAA, Pasching, Austria) for CV-1 and 20% FBS for MTH52c, ZMTH3 and CT1258 at 37 °C under 5% CO_2_. STSA-1 cells were cultivated in minimum essential medium (MEM) with Earle’s salts supplemented with 2 mM glutamine, 50 U/mL penicillin G, 50 µg/mL streptomycin, 1 mM sodium pyruvate, 0.1 mM nonessential amino acids (MEM-C) and 10% FBS.

### 2.3. Virus Strains

Vaccinia virus strain LIVP 6.1.1 was derived from LIVP (Lister strain, Institute of Viral Preparations, Moscow, Russia). The sequence analysis of LIVP 6.1.1 revealed the presence of different mutations in the *J2R* gene locus encoding the thymidine kinase [7]. GLV-5b451 virus was derived from the oncolytic vaccinia virus LIVP 6.1.1 by inserting the glaf-2 gene encoding the GLAF-2 antibody under the control of the vaccinia virus synthetic early-late (SEL) promoter [9] into the *J2R* locus [25].

### 2.4. Cell Viability Assay

MTH52c, ZMTH3, CT1258 or STSA-1 cells were seeded in 24-well plates (Greiner Bio-One, Frickenhausen, Germany). After 24 h in culture, the cells were infected with either LIVP 6.1.1 or GLV-5b451 using multiplicities of infection (MOI) of 0.1. The cells were incubated at 37 °C for 1 h, then the infection medium was removed and subsequently the cells were incubated in fresh growth medium. The amount of viable cells after infection was measured using 3-(4,5-dimethylthiazol-2-yl)-2,5-diphenyltetrazolium bromide (MTT) (Sigma, Taufkirchen, Germany). At 24, 48, or 72 h post infection of cells, the medium was replaced by 0.5 mL MTT solution at a concentration of 2.5 mg/mL MTT dissolved in DMEM without phenol red and incubated for 2 h at 37 °C in a 5% CO_2_ atmosphere. After removal of the MTT solution, the color reaction was stopped by adding 1 N HCl diluted in isopropanol. The optical density was then measured at a wavelength of 570 nm in a Tecan Sunrise Remote microplate reader (Tecan, Männedorf, Switzerland). Uninfected cells were used as reference and were considered as 100% viable.

### 2.5. Viral Replication

For the viral replication assay, MTH52c, ZMTH3, CT1258 or STSA-1 cancer cells were infected with LIVP 6.1.1 or GLV-5b451 at an MOI of 0.1. After one hour of incubation at 37 °C, the infection medium was removed and replaced by fresh growth medium. After 1, 24, 48, 72 and 96 h, the cells and supernatants were harvested. Following three freeze-thaw cycles and three times sonication (3 × 30 s), serial dilutions of the supernatants and lysates were titered by standard plaque assay on CV-1 cells. All samples were measured in triplicate.

### 2.6. ELISA

For determination of relative canine VEGF concentrations in MTH52c, ZMTH3, CT1258 or STSA-1 cell culture supernatants, 5 × 10^6^ cells were cultured in DMEM or MEM containing 10% FBS. Cell culture supernatants were collected at 72 and 96 h and stored at −20 °C. Concentrations of VEGF were determined by VEGF ELISA kit (Thermo Scientific, Rockford, IL, USA) developed for detection of human VEGF (cross-reacts approximately 67% to recombinant canine VEGF; R&D Systems, Inc., Minneapolis, MN, USA, catalog number DVE00, page 11, http://www.rndsystems.com/), in accordance with the manufacturer’s directions.

For the determination of the affinity and cross-reactivity of GLAF-2 to VEGF from different species, an ELISA was performed. For this purpose recombinant canine (1603-CV-010, R&D Systems Inc., Minneapolis, MN, USA), murine (V4512, Sigma-Aldrich, St. Louis, MO, USA) and human (V7259, Sigma-Aldrich) VEGF proteins were pre-coated at a concentration of 100 ng/well in 96-well ELISA plates and incubated overnight at 4 °C. The wells were washed once with bidest. water and twice with PBS/0.05% Tween (PBST) and blocked with 100 µL 1% w/v Blocker^TM^ Casein in PBS (Pierce, 37528) for 2 h at 37 °C. After washing the wells four times with PBST, wells were incubated with seven two-fold dilution series of GLAF-2 (2000 to 31.25 ng/mL, GenScript, *E.coli* expressed and purified tag-free) for 1 h at room temperature (RT). PBS was used as a negative control. The wells were washed again and incubated with a polyclonal rabbit anti-GLAF-2 antibody (1:1000, GenScript, Piscataway, NJ, USA) for 1 h at RT. After washing the wells were incubated with a HRP-conjugated polyclonal goat anti-rabbit IgG (H+L) (170-6515, Biorad, 1:5000) for 1 h at RT. The plate was washed and staining was developed using 3,3′,5,5′-Tetramethylbenzidine (T0440, Sigma-Aldrich) and stopped with 2 N HCl. The ELISA was read at a wavelength of 450 nm.

### 2.7. Western Blot Analysis

For detection of virus encoded proteins, MTH52c, ZMTH3, CT1258 or STSA-1 cells were harvested and resuspended in SDS sample buffer at 1, 24, 48, 72, or 96 h post virus infection (hpvi). Samples were separated by 10% SDS-Polyacrylamide gel electrophoresis and subsequently transferred onto a nitrocellulose membrane (Whatman GmbH, Dassel, Germany). After blocking in 5% skim milk in PBS, the membrane was incubated with rabbit anti-G6 antibody (affinity-purified polyclonal antibody raised against a GLAF-2 peptide, GenScript, Piscataway, NJ, USA) for detection of scAb GLAF-2 or polyclonal rabbit anti-vaccinia virus antibody (ab35219 Abcam, Cambridge, UK). The primary antibodies were detected using horseradish peroxidase-conjugated anti-rabbit (ab6721, Abcam, Cambridge, UK) secondary antibody, followed by enhanced chemiluminescence detection.

### 2.8. Vaccinia Virus-Mediated Therapy of STSA-1 Xenografts

Tumors were generated by implanting 2 × 10^6^ canine soft tissue sarcoma STSA-1 cells subcutaneously into the right hind leg of 6- to 8-week-old female nude mice (Hsd: Athymic Nude-*Foxn1*^nu^; Harlan, Netherlands). Tumor growth was monitored at least one time weekly in two dimensions using a digital caliper. Tumor volume was calculated as [(length × width^2^)/2]. When tumor volume reached approximately 300–1000 mm^3^ (STSA-1) groups of mice were injected either with 1 × 10^7^ pfu of GLV-5b541 or LIVP 6.1.1 viruses or PBS (control) into the tail vein. The significance of the results was calculated by Student’s *t*-test. Results are displayed as means +/− standard deviation (SD). *p* values <0.05 were considered significant. Mice were monitored for change in body weight and signs of toxicity.

### 2.9. Histology and Microscopy

For histological studies, tumors were excised and snap-frozen in liquid nitrogen, followed by fixation in 4% paraformaldehyde/PBS at pH 7.4 for 16 h at 4 °C. After dehydration in 10% and 30% sucrose (Carl Roth, Karlsruhe, Germany) specimens were embedded in Tissue-Tek O.C.T. (Sakura Finetek Europe B.V., Alphen aan den Rijn, The Netherlands). Tissue samples were sectioned (10 mm thickness) with the cryostat 2800 Frigocut (Leica Microsystems GmbH, Wetzlar, Germany). Labeling of tissue sections was performed as described in detail elsewhere [25]. In this case, VACVs were labeled using polyclonal rabbit anti-vaccinia virus (anti-VACV) antibody (Abcam, Cambridge, UK), which was stained using Cy2-conjugated donkey anti-rabbit secondary antibodies obtained from dianova (Hamburg, Germany). Endothelial blood vessel cells were stained with a hamster monoclonal anti-CD31 antibody (Merck Millipore KGaA, Darmstadt, Germany, MAB1398Z) and a DyLight649-conjugated goat anti hamster secondary antibody from dianova.

The fluorescence-labeled preparations were examined using a TCS SP2 AOBS confocal laser microscope (Leica Microsystems GmbH, Wetzlar, Germany) equipped with the LCS 2.16 software (102461024 pixel RGB-color images). Digital images were processed with Photoshop 7.0 (Adobe Systems, Mountain View, CA, USA).

### 2.10. Measurement of Blood Vessel Density and Fluorescence Intensity of the CD31 Signal in the Tumor Tissue

Blood vessel density was measured in digital images (× 20 magnification) of CD31-labelled 10-mm-thick tumor cross-sections using a TCS SP2 AOBS confocal laser microscope (Leica Microsystems GmbH, Wetzlar, Germany) equipped with the LCS 2.16 software (1024 × 1024 pixel RGB-color images). Twenty-four images per virus-treated tumor (3 sections of each tumor and 8 images per section) and 12 images per control PBS tumor were analyzed. Exposure time was adjusted for all images using Photoshop 7.0. All blood vessels were counted to obtain the vessel density per section.

Fluorescence intensity of the CD31-labeling was measured using ImageJ (version 1.44p) software as described in detail elsewhere [17]. RGB-images were converted into 8-bit gray scale images using Photoshop 7.0. ImageJ was used to adjust the threshold of all images and to analyze the number of particles per area. The fluorescence intensity of the CD31-labelling represents the average brightness of all vessel-related pixels.

### 2.11. Statistical Analysis

The statistical significance of differences between groups of animals was analyzed using unpaired or paired Student’s *t*-test. *t*-tests were done in “R” (Retrieved from http://www.r-project.org). *p*-value <0.05 was considered significant.

## 3. Results

### 3.1. VEGF Expression in Different Canine Cancer Cell Lines under Cell Culture Conditions

**Figure 1:**
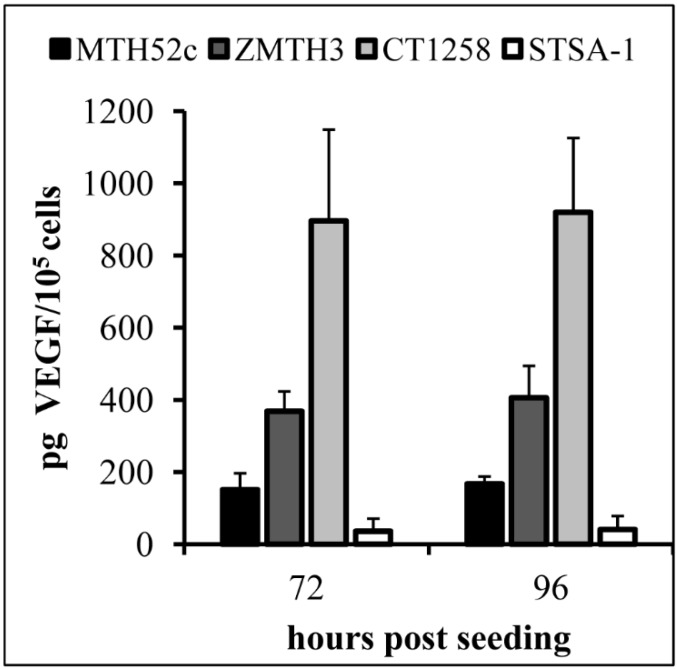
VEGF expression in MTH52c, ZMTH3, CT1258 or STSA-1 canine cancer cells under cell culture conditions. Each value represents the mean (*n* = 3) +/− standard deviations (SD).

VEGF is a potent mediator of both angiogenesis and vasculogenesis in dogs and has been proposed as a prognostic indicator in several types of canine cancer [10,13,26]. Therefore, we investigated the question, if canine cancer cells produce VEGF under cell culture conditions (Figure 1).

VEGF concentrations were determined using a VEGF ELISA kit (Thermo Scientific, Rockford, IL, USA) in accordance with the manufacturer’s directions. VEGF levels in the supernatant of MTH52c, ZMTH3, CT1258 or STSA-1 cells were determined at 72 and 96 h. The highest concentration of VEGF was found in the supernatants of CT1258 cells, 895.71 ± 253.05 pg/10^5^ cells (72 h) and 919.94 ± 206.18 pg/10^5^ cells (96 h). The results revealed that CT1258 cells produced about 25-fold more canine VEGF compared to STSA-1 cells at these two different time points. In summary, all tested cells produced VEGF constitutively under cell culture conditions.

### 3.2. Analysis of the Oncolytic Potential of GLV-5b451 Virus against Different Canine Cancer Cell Lines by MTT Assay

**Figure 2:**
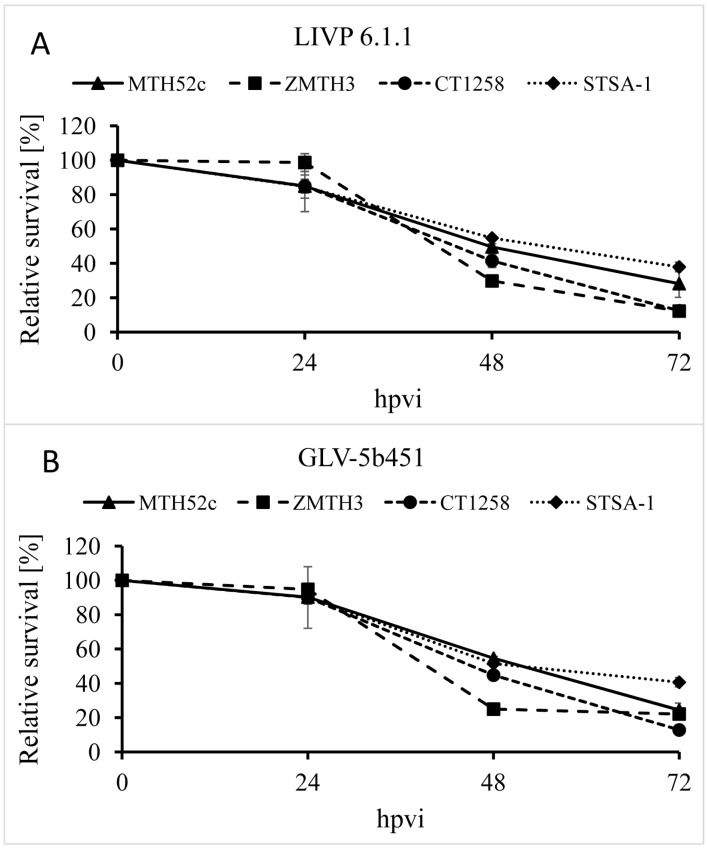
Relative survival of MTH52c, ZMTH3, CT1258 or STSA-1 canine cancer cells after LIVP 6.1.1 or GLV-5b451 infection at an MOI of 0.1. Viable cells were detected using 3-(4,5-dimethylthiazol-2-yl)-2,5-diphenyltetrazolium bromide (MTT). Mean values (*n* = 3) and standard deviations are shown as percentages of respective controls. The data represent two independent experiments.

The oncolytic effect of GLV-5b451 and the parental strain LIVP 6.1.1 (control) against four different canine cancer cell lines including mammary carcinoma (MTH52c), mammary adenoma (ZMTH3), prostate carcinoma (CT1258) and soft tissue sarcoma (STSA-1) cells was examined. For this purpose, the cells were seeded three days prior to infection in 24-well plates and then were infected with either GLV-5b451 or LIVP 6.1.1 at a multiplicity of infection (MOI) of 0.1. The cell viability was analyzed at 24, 48 and 72 h post-virus-infection (hpvi) by MTT-assays (Figure 2). At this MOI, both viruses were cytotoxic, resulting in at least 60% killing capacity to all the cell lines over 72 hpvi.

The data demonstrated that GLV-5b451 and LIVP 6.1.1 efficiently infected and destroyed different canine cancer cells under these cell culture conditions. There was no statistically significant difference in the number of viable cells between the two virus strains.

### 3.3. GLV-5b451 Virus Efficiently Replicates in MTH52c, ZMTH3, CT1258 or STSA-1 Canine Cancer Cells

**Figure 3:**
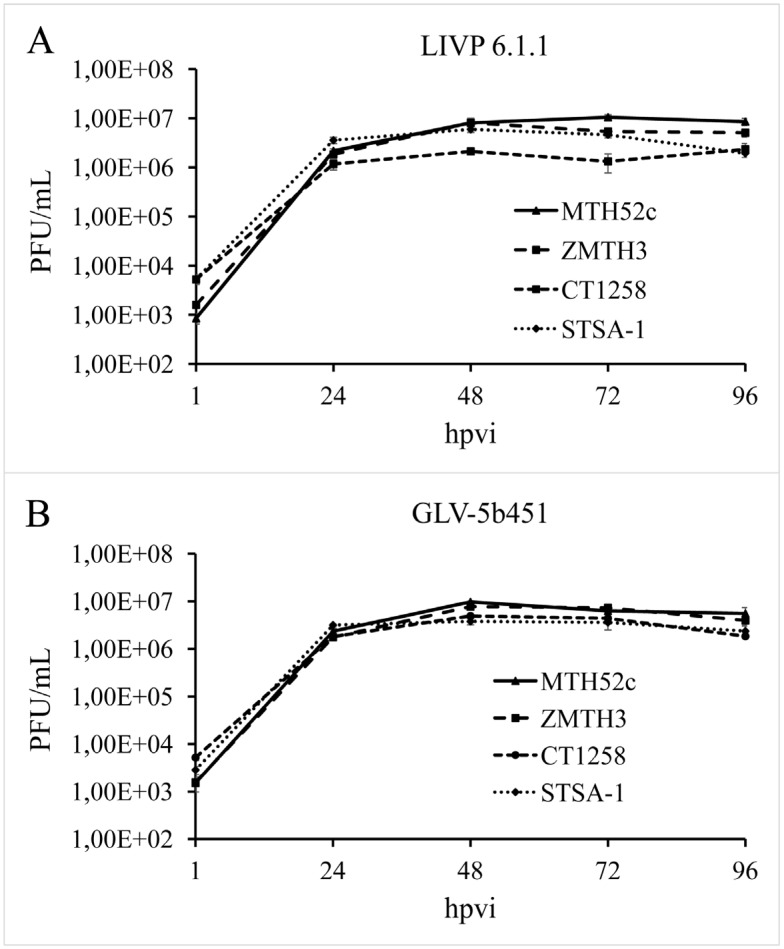
Replication capacity of the vaccinia virus strains LIVP 6.1.1 (**A**) and GLV-5b451 (**B**) in different canine cancer cell lines. For the viral replication assay, MTH52c, ZMTH3, CT1258 or STSA-1 cells grown in 24-well plates were infected with either LIVP 6.1.1 or GLV-5b451 at an MOI of 0.1. Cells and supernatants were collected for the determination of virus titers at various time points. Viral titers were determined as pfu per ml in triplicates by standard plaque assay in CV-1 cell monolayers. Averages plus standard deviation are plotted. The data represent three independent experiments.

In order to test the efficiency of virus replication, MTH52c, ZMTH3, CT1258 or STSA-1 cells were infected with either GLV-5b451 or LIVP 6.1.1 at an MOI of 0.1. In this experimental setting LIVP 6.1.1 was used as a control. Standard plaque assays were performed for all samples to determine the viral titers at different time points during the course of infection (Figure 3). The maximum viral titers were determined for GLV-5b451 (9.73 × 10^6^ pfu/mL) in MTH52c cells at 48 hpvi (Figure 3A) and for LIVP 6.1.1. (1.05 × 10^7^ pfu/mL) at 72 hpvi (Figure 3B). In addition, efficient viral replication (>100-fold titer increase from 24 to 96 hpvi) was observed in all tested cell lines (Figure 3).

The data demonstrated that replication efficiency of GLV-5b451 and LIVP 6.1.1 was not significantly different between the four tested tumor cell types.

### 3.4. Production of GLAF-2 Antibody in GLV-5b451-Infected Canine Cancer Cells

**Figure 4:**
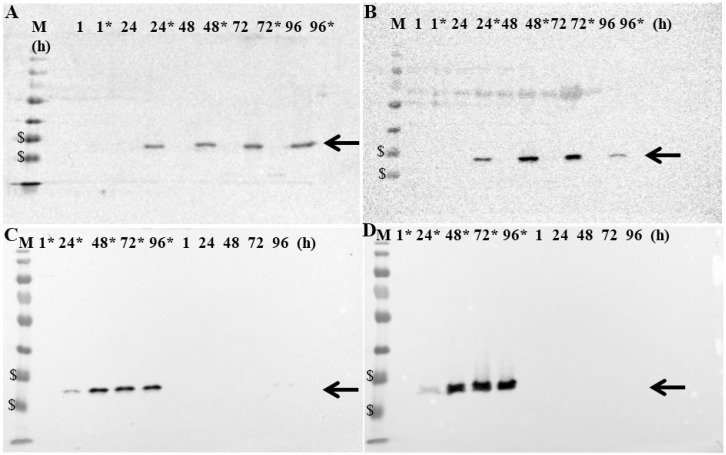
Expression of GLAF-2 protein in different canine cancer cells: (**A**) MTH52c; **(B**) ZMTH3; (**C**) CT1258 and (**D**) STSA-1. Western blot analysis of GLV-5b451-infected (MOI of 1.0; lines marked by *) or uninfected canine cancer cells. Protein fractions from cell lysates were isolated at 1, 24, 48, 72 and 96 h post virus infection and separated by SDS-PAGE. Western blot analysis was performed as described in material and methods. The position of GLAF-2 protein is marked by black arrow. M: PageRuler Prestained Protein Ladder # 26616 (Thermo Scientific, Bonn, Germany). The positions of the 35 and 25 kDa proteins are marked by $ symbol.

MTH52c, ZMTH3, CT1258 or STSA-1 cells were infected with GLV-5b451 at an MOI of 1.0 in 24-well plates. At 1, 24, 48, 72 and 96 hpvi, cells were harvested and analyzed by Western Blot using anti-G6 (GLAF-2) or anti-vaccinia virus (VV) antibodies, respectively (Figure 4, Supplementary Figure S1).

Uninfected cells were harvested under the same conditions and were used as controls. The data clearly demonstrated that GLV-5b451-infected cells expressed the GLAF-2 protein (Figure 4) of expected size (27 kDa). In addition, the GLAF-2 expression correlated well with the expression of vaccinia virus specific proteins (Supplementary Figure S1). Under these experimental conditions we found the highest GLAF-2 production in the GLV-5b451-infected STSA-1 cells. Therefore, we have chosen these cells for further studies.

### 3.5. The GLAF-2 Antibody Specifically Recognizes Canine VEGF

**Figure 5:**
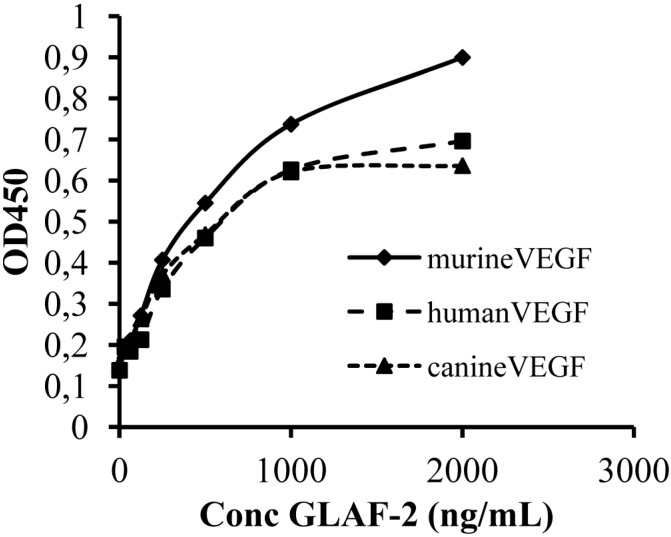
Interactions of purified GLAF-2 antibodies with canine, murine, and human VEGFs. Affinity and cross reactivity of GLAF-2 was demonstrated by ELISA. Equal concentrations of canine, murine, or human VEGF (100 ng/well) were coated on ELISA plates. Seven two-fold dilutions of purified GLAF-2 proteins ranging from 2000 ng/mL to 31.3 ng/mL were incubated with canine, murine and human VEGFs. PBS was used as negative control. For further ELISA experimental conditions see material and methods. ODs obtained for various concentrations of GLAF-2 against canine, murine and human VEGF were plotted. ELISA was repeated in three independent experiments. Each value represents the mean (*n* = 3) +/− standard deviations (SD).

Since until now the affinity of GLAF-2 to canine (ca) VEGF has not been characterized yet, we tested the ability of GLAF-2 antibody to bind recombinant canine VEGF by ELISA. In these experimental settings we used murine (m) as well as human (h) VEGF as controls. The data demonstrated that the GLAF-2 antibody can recognize and bind caVEGF with similar efficiency like the human and murine VEGF controls (Figure 5).

### 3.6. A Single Systemic Administration of GLV-5b451 Significantly Regresses Growth of STSA-1 Derived Tumors in Nude Mice

Twenty-one female nude mice at an age of 6–8 weeks were implanted with 2 × 10^6^ STSA-1 cells. Four weeks post implantation, all mice developed tumors with volumes of 300 to 1000 mm^3^. Animals were separated into three groups (*n* = 7) and were injected with a single dose of GLV-5b451, LIVP 6.1.1 (1 × 10^7^ pfu in 100 µL PBS) or PBS (100 µL) intravenously (i.v.) into the lateral tail vein. LIVP 6.1.1, a non-GLAF-2 expressing parental virus strain of GLV-5b451 virus, was used as an additional control. Tumor size was measured at least one time per week.

**Figure 6:**
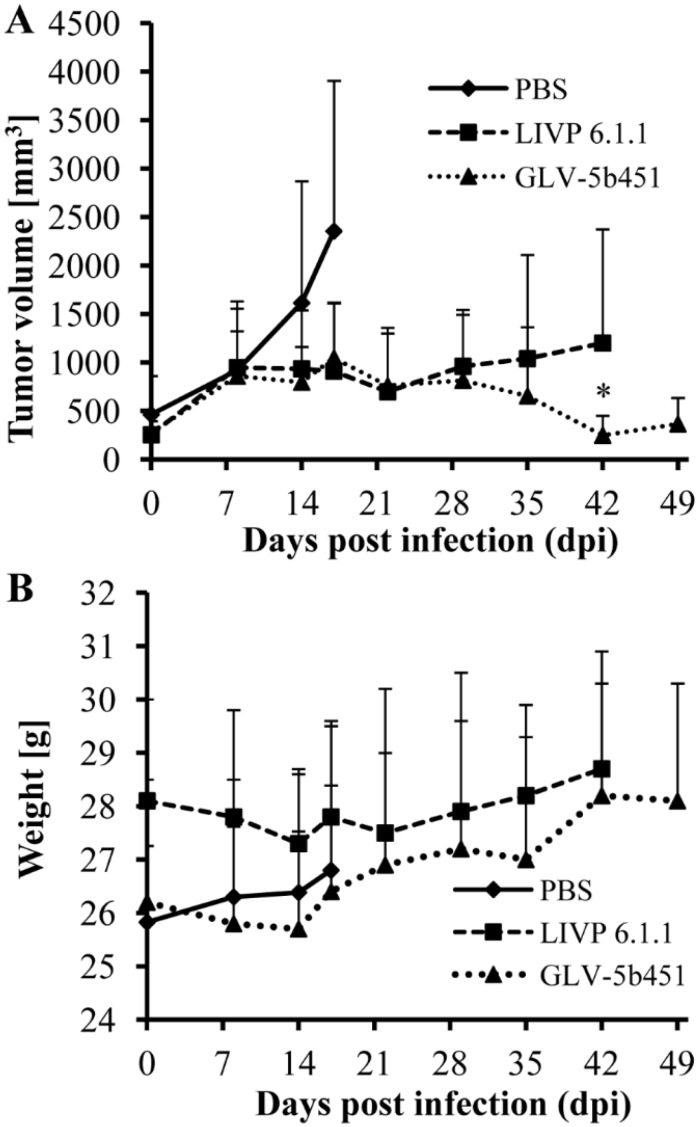
Effects on tumor growth (**A**) and body weights (**B**) of virus- and mock-treated STSA-1 xenografted mice. (A) Groups of STSA-1-tumor-bearing nude mice (*n* = 7) were either treated with a single dose of 1 × 10^7^ pfu GLV-5b451 or LIVP 6.1.1 or with PBS (mock control). Statistical analysis was performed with a paired Student’s *t*-test (* *p* < 0.05); (B) Relative mean weight changes of STSA-1 cell xenografted mice after virus or PBS treatment. The data are presented as mean values +/− SD.

As shown in Figure 6A, the virus treatment led to significant differences in tumor growth between PBS controls and all virus-treated mice. Due to excessive tumor burden (>3000 mm^3^), all animals in the control PBS group were euthanized after 17 days post virus injection (dpvi). At this time point, there was no significant difference between the two virus treated groups (GLV-5b451 *vs.* LIVP 6.1.1; *p* = 0.5761). Interestingly, the GLV-5b451 injection led to significantly better inhibition of the tumor growth (* *p* = 0.04981) compared to the LIVP 6.1.1-treated animals on 42 dpvi. Moreover, one of seven mice of the LIVP 6.1.1-treated group developed tumor with volume greater than 3000 mm^3^ and had to be euthanized.

The toxicity of the GLV-5b451 virus was determined by monitoring the weight change of mice over time (Figure 6B). All virus-treated mice showed stable mean weight over the course of studies. There were no signs of virus-mediated toxicity.

### 3.7. Virus Distribution, GLAF-2 Production and the Tumor Vascularization in STSA-1 Tumor-Bearing Nude Mice after Virus Injection

To investigate the possible reasons for different outcome of the virotherapy, we started a second therapeutic experiment in which virus distribution and tumor vascularization after injection with GLV-5b451, LIVP 6.1.1 or PBS were compared. For this purpose, groups of three tumor-bearing mice were injected either with 1 × 10^7^ pfu of GLV-5b451, LIVP 6.1.1 or with PBS. The virus distribution was analyzed at day 17. We have chosen this experimental time point for our analysis because first the inhibition of the tumor growth of all GLV-5b451 virus-treated mice was observed after 17 dpvi (Figure 6A). Viral titers were determined by standard plaque assays on CV-1 cells using aliquots of homogenized organs or tumor tissues (Table 1). The highest viral titers from 8.4 × 10^5^ to 1.44 × 10^7^ pfu/g were detected in primary tumors of virus-treated mice. In the tumor tissues no significant difference of the virus titers between LIVP 6.1.1- and GLV-5b451-injected groups was detected. Only very few virus particles were detected in the healthy organs, such as spleens, livers and lungs, of the virus-injected mice (Table 1). These data clearly show that both viruses can preferentially replicate in the tumor tissue only.

**Table 1:** Biodistribution of either LIVP 6.1.1- or GLV-5b451-injected STSA-1 xenografts at 17 dpvi.

PFU per Gram (g) of Organ or Tumor Tissue	Tumor	Spleen	Lung	Liver
**LIVP 6.1.1-Injected Group**				
Mouse No. 401	9.6E+06	n.d.	2.0E+02	7.6E+03
Mouse No. 418	8.4E+05	n.d.	2.8E+02	8.0E+02
Mouse No. 419	1.44E+07	9.6E+02	1.8E+03	4.0E+02
**GLV-5b541-Injected Group**				
Mouse No. 403	1.1E+07	2.8E+03	n.d.	1.6E+03
Mouse No. 414	2.4E+06	4.0E+02	4.8E+02	8.0E+02
Mouse No. 409	7.2E+06	2.2E+03	4.8E+02	5.6E+02

The data were determined by standard plaque assay on CV-1 cells using aliquots of the homogenized organs and were displayed as mean pfu per gram of organ or tissue. For each organ, two aliquots of 0.1 mL were measured in triplicates. n. d.: not detected (detection limit *<*10 pfu/organ).

**Figure 7:**
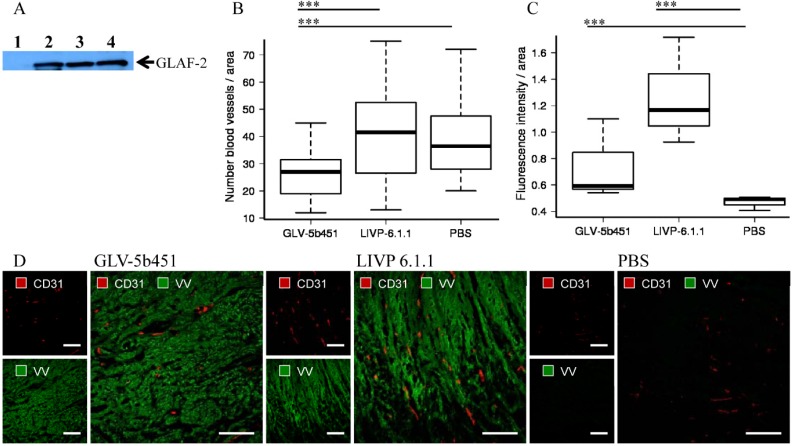
Presence of the scAb GLAF-2 (**A**) and analysis of vascular density in tumor tissues of virus- or PBS-injected STSA-1 xenograft mice at 17 dpvi (**B**–**D**). (A) Western blot analysis of GLV-5b451-infected STSA-1 tumors at 17 dpvi (lines 2–4). Line 1: Lysate of a LIVP 6.1.1-infected STSA-1 tumor (negative control). Each sample represents an equivalent of 1.5 mg tumor mass; (B,C) Visualization and analysis of vascular density using CD31 immunohistochemistry in LIVP 6.1.1, GLV-5b451 or PBS-treated tumors: (B) The vascular density was measured in CD31-labeled tumor cross-sections (*n* = 3 mice per group, 24 images per virus-injected mouse or 12 images per control PBS mouse) and presented as mean values +/− SD. (*** *p* < 0.001; ** *p* < 0.01;* *p* < 0.05 Student’s *t*-test); (**C**) Fluorescence intensity of the CD31 signal of blood vessels. The fluorescence intensity of the CD31-labelling represents the average brightness of all vessel-related pixels and was determined as described in materials and methods. Shown are the mean values +/− standard deviations. Statistical analysis was performed with a two-tailed unpaired Student’s *t*-test (*** *p* < 0.001; ** *p* < 0.01; * *p* < 0.05); (D) Representative tumor sections labeled with anti-CD31 antibody (red) and anti-vaccinia virus (VV) antibody (green). Scale bars: 150 mm.

To test the effect of the GLAF-2 antibody on tumor angiogenesis, we first analyzed intra-tumoral GLAF-2 expression in GLV-5b451-treated STSA-1 tumors. The presence of GLAF-2 antibodies was observed in tumor tissues of all GLV-5b451-treated mice but not in control animals (Figure 7A).

In addition, tumor angiogenesis was assessed by CD31 immunostaining and microvessel density analysis. For this purpose, CD31-labelled cross sections of tumors from LIVP 6.1.1-, GLV-5b451- and PBS-treated mice were compared by fluorescence microscopy at day 17 after treatment (Figure 7B–D). Thereby we observed the significantly decreased amount of CD31-positive vessels in GLV-5b451-infected tumors when compared to that of LIVP 6.1.1- and PBS-injected control tumors (GLV-5b451 *vs.* LIVP 6.1.1 *** *p* = 0.00000213; GLV-5b451 *vs.* PBS *** *p* = 0.000000000947; Figure 7B).

The fluorescence intensity (FI) of the CD31 signal of blood vessels was additionally measured as described in material and methods. In this experimental setting, FIs of vessel-related pixels of LIVP 6.1.1- and GLV-5b451-tumors were significantly increased in comparison to PBS-injected control tumors (Figure 7C). This means that the virus colonization led to an upregulation of CD31 protein by vascular cells in the tumor tissues.

In summary, the application of the GLV-5b451-virus producing scAb GLAF-2 led to a significant inhibition of the blood vessel development in comparison to LIVP 6.1.1 and PBS-injected controls (Figure 7).

## 4. Discussion

In the current study, we investigated the oncolytic efficacy of the recombinant vaccinia virus strain GLV-5b451 expressing the anti-VEGF single-chain antibody GLAF-2 in four different canine cancer cell lines. The results showed that GLV-5b451 was able to effectively infect, replicate in and lyse all tested canine cells. The virus infection led to efficient expression of GLAF-2 protein in all four canine cell lines (Figure 4 and Supplementary Figure S1B) without any reduction of effectiveness in its oncolytic potential compared with the parental LIVP 6.1.1 virus (Figure 2 and Figure 3). In addition, we have also shown for the first time that GLAF-2 can specifically bind to canine VEGF with a similar efficacy like human VEGF (Figure 5). VEGF is a strong bioactive agent involved in processes of tumor angiogenesis and vascular permeability [27,28] and therefore a promising target for cancer therapy. VEGF or its pathway have been successfully targeted with antibodies and small molecules [14,15]. One of the best-characterized strategies is the use of a humanized monoclonal antibody (mAb) bevacizumab (avastin) to neutralize the human VEGF [29]. However, bevacizumab did not provide a significant benefit in cancer patients as a monotherapy [30], but conferred survival benefit in combination with chemotherapy or immunotherapy [31].

The lack of efficacy of bevacizumab after systemic treatment in cancer patients may be at least attributable to the poor penetration of this antibody into the tumor tissue and metastases as well as rapid clearance from the circulation after systemic administration [32]. Interestingly, we have recently reported that treatments with recombinant vaccinia virus strains (VACV) expressing anti-VEGF antibodies (GLAF-1 or GLAF-2) led to enhanced tumor growth inhibition and vascular disruption in different xenograft models [8,9,17,25,33]. These data demonstrated, that the oncolytic VACV not only preferentially targets and destroys tumor cells but also mediates local production of therapeutic antibodies in colonized tumors [9,17,25]. A potential explanation for this therapy success could be that the combination of oncolytic vaccinia virus and anti-VEGF antibody allows a better delivery and/or better interaction between the antibody and VEGF in the tumor tissue when compared to systemic delivered antibody alone. In addition, we have already found, that the presence of GLAF-2 antibody in GLV-5b451-infected feline xenograft tumors led to significant reduction of the VEGF levels when compared to the control tumors [25]. Moreover, a decrease of VEGF levels in tumor tissues after treatment with an anti-VEGF antibody delivered by oncolytic vaccinia virus was also observed in human xenograft models [9,33]. On the other hand, Hiley et al, demonstrated that overexpression of VEGF could also enhance vaccinia virus infection within tumor tissue *in vivo* after systemic delivery [34]. These results highlighted the importance of VEGF expression for the initial viral infection. Interestingly, VEGF can often be found in elevated concentrations in blood and tumor of canine cancer patients correlating with an unfavorable prognosis in several tumor types [10,13,26]. Such tumors may be potential targets for VACV dependent virotherapy. All these facts advance VACVs expressing anti-VEGF antibodies as promising therapeutic agents for inhibition of angiogenesis via VEGF reduction in canine cancer patients.

In our STSA-1 xenograft model, the vascular density of GLV-5b451 tumors was significantly decreased in comparison to that of LIVP 6.1.1- and PBS-treated-tumors at 17 dpvi (Figure 7B). This could also be evidence that GLAF-2-presence “normalizes” the abnormal vasculature of tumors, resulting in improved survival of the treated animals (Figure 6A). Moreover a number of anti-VEGF agents have been shown to normalize tumor vessel structure and function in animal models and patients [31]. In addition, after virus infection, significant increased expression of CD31 was observed in the tumor vasculature, but not in the PBS control tumors (Figure 7C). This means that the virus colonization led to an upregulation, of CD31 protein, which mediates transendothelial migration of immune cells to sites of infection as shown in several previous studies [17,24,35]. An activated endothelium that is characterized by vascular hyperpermeability might also be one additional reason for a better delivery of therapeutic antibodies in the virus-infected tumors in comparison to that of systemic delivery alone [9].

After comparing viruses expressing anti-VEGF antibodies, we found that treatment with GLV-5b451 expressing GLAF-2 led to significantly better inhibition of the tumor growth than GLV-1h109 expressing GLAF-1 in the same STSA-1-xenograft model [17]. In addition, in current study, we demonstrated that treatment with GLV-5b451 not only inhibited tumor growth, but actually reduced tumor volume by 90% within 42 dpvi. Moreover, we were able to show complete eradication of tumors in 30% of mice treated with GLV-5b451. These data are different from data we reported after GLV-1h109 treatment [17]. A better therapeutic effect of GLV-5b451 in comparison to GLV-1h109 might be due to the backbone virus, since there is no evidence for functional differences between GLAF-1 and GLAF-2 antibodies.

## 5. Conclusions

Taken all together, the oncolytic vaccinia virus strain GLV-5b451 may be a better oncolytic therapy agent compared to GLV-1h109 or LIVP6.1.1 and a promising candidate for therapy of canine cancer patients with CSTS.

## Supplementary Files

Supplementary File 1
