# Supplementary material for: Preclinical Testing Oncolytic Vaccinia Virus Strain GLV-5b451 Expressing an Anti-VEGF Single-Chain Antibody for Canine Cancer Therapy
Source: Viruses. 2015 Jul 20;7(7):4075–92. doi: 10.3390/v7072811 (PMC4517140; doi:10.3390/v7072811)
Supplement: Supplementary File 1 [file viruses-07-02811-s001.pdf]

Supplementary Information

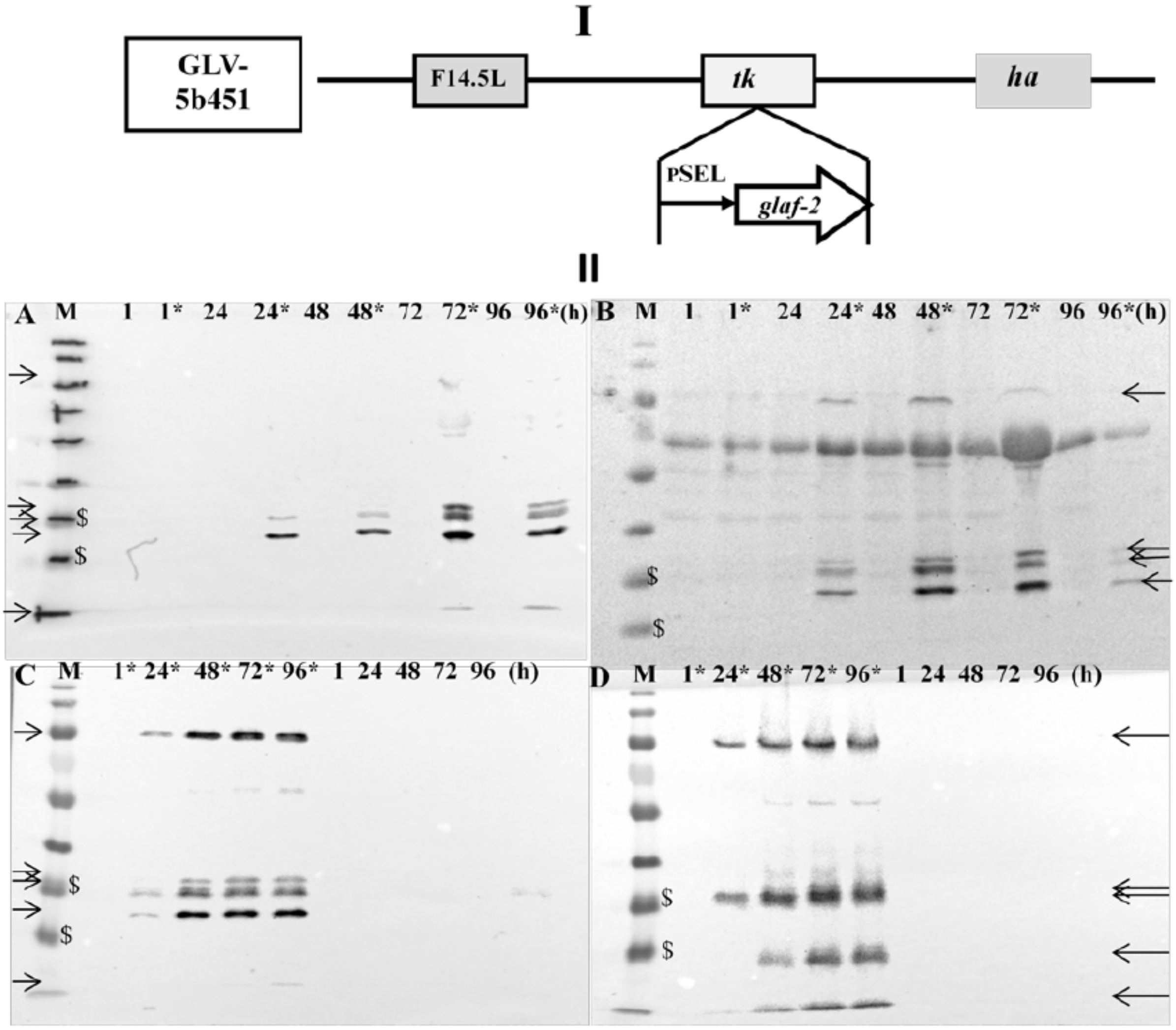

Figure S1. Expression of vaccinia virus mediated proteins in canine cancer cells. (**I**) Schematic representation of GLV-5b451. F14.5L: *vaccinia virus* F14.5L *gene* product; *tk*: thymidine kinase; *ha*: hemagglutinin; pSEL: synthetic early-late promoter; (**II**) Expression of vaccinia virus specific proteins in different canine cancer cells: (**A**) MTH52c; (**B**) ZMTH3; (**C**) CT1258 and (**D**) STSA-1. Western blot analysis of GLV-5b451-infected (MOI of 1.0; lines marked by *) or uninfected canine cancer cells. Protein fractions from cell lysates were isolated at 1, 24, 48, 72 and 96 h post virus infection and separated by SDS-PAGE. Western blot analysis was performed as described in material and methods. The positions of virus specific proteins are marked by black arrows. M: PageRuler Prestained Protein Ladder # 26616 (Thermo Scientific, Bonn, Germany). The positions of the 35 and 25 kDa proteins are marked by $ symbol.

© 2015 by the authors; licensee MDPI, Basel, Switzerland. This article is an open access article distributed under the terms and conditions of the Creative Commons Attribution license (http://creativecommons.org/licenses/by/4.0/).
